# Chronic Alcohol Consumption Promotes Diethylnitrosamine-Induced Hepatocarcinogenesis via Immune Disturbances

**DOI:** 10.1038/s41598-017-02887-7

**Published:** 2017-05-31

**Authors:** Guoxiu Yan, Xuefu Wang, Cheng Sun, Xiaodong Zheng, Haiming Wei, Zhigang Tian, Rui Sun

**Affiliations:** 1 Institute of Immunology and CAS Key Laboratory of Innate Immunity and Chronic Disease, School of Life Sciences and Medical Center, University of Science and Technology of China, Hefei, Anhui 230027 China; 2 Hefei National Laboratory for Physical Sciences at Microscale, Hefei, Anhui 230027 China

## Abstract

Chronic alcohol consumption increases the risk of hepatocellular carcinoma (HCC). However, little is known about the potential immunological mechanisms by which ethanol affects tumor progression. Here, adult male mice were administered multiple doses of diethylnitrosamine (DEN). Four and a half months later, the DEN-treated mice were placed on a liquid Lieber-DeCarli control diet or diet containing 5% ethanol for 2.5 months. At the end of the study, liver tissue samples were obtained to analyze pathology, gene expression, and hepatic mononuclear cells (MNCs). Results showed that ethanol feeding exacerbates the progression of hepatic tumors (characterized by the ratio of liver weight to body weight, and the tumor volume and diameter) in DEN-treated mice. Mechanistically, chronic alcohol consumption decreased the number of antitumor CD8^+^ T cells but increased the number of tumor-associated macrophages (TAMs) in the liver in DEN-initiated tumorigenesis. Besides, TAMs were prone to be M2 phenotype after alcohol consumption. Moreover, chronic alcohol consumption aggravated inflammation, fibrosis, and epithelial-mesenchymal transition (EMT) in the pathological process of HCC. These data demonstrate that chronic alcohol consumption exacerbates DEN-induced hepatocarcinogenesis by enhancing protumor immunity, impairing antitumor immunity and aggravating hepatic pathological injury. Targeting the immune system is a potential therapeutic regimen for alcohol-promoted HCC.

## Introduction

Hepatocellular carcinoma (HCC) is a primary hepatic malignancy, the third leading cause of cancer-related deaths and the fifth most common tumor worldwide. HCC predominantly occurs in the context of chronic liver disease, which is usually contributed by hepatitis B or hepatitis C infection and chronic alcohol consumption. The highest incidence rates of HCC have been reported in East Asia, particularly in China, where people are susceptible to HBV infection^1^. Epidemiology has characterized alcoholic liver disease (ALD)-associated cirrhosis as a medium-high risk factor for HCC^2^.

The establishment of HCC is a multistage process that involves inflammation, cirrhosis, and epithelial-mesenchymal transition (EMT). Alcohol abuse and chronic HBV or HCV infection leads to chronic hepatitis accompanied by inflammation, oxidative stress, and metabolic disorders. Over time, hypoxia, genetic instability, and immune evasion become key features of the liver microenvironment. In general terms, patients with chronic hepatitis develop fibrosis and cirrhosis and then HCC; however, certain patients are at high risk of developing HCC in the absence of advanced cirrhosis^3^. Chronic alcohol consumption leads to ALD, including steatosis, alcoholic steatohepatitis, and cirrhosis^4^. Chronic alcohol consumption contributes to the occurrence of HCC through metabolic disorders, such as acetaldehyde formation, reduced antioxidants, hypomethylation, iron overload, and reduced retinoic acid^5^. However, the existing evidence of the effect of chronic alcohol consumption on HCC development using different models is contradictory^6–9^.

The liver is increasingly regarded as an immunological organ and is enriched in Kupffer cells, γδ T cells, natural killer (NK) cells, and natural killer T (NKT) cells^10^. Immune cells exert immunosurveillance in the early stages by removing premalignant and fully transformed cells. Immunosurveillance depends on NK cells, CD8^+^ cytotoxic T cells (CTLs), and various cytokines^11^. In the advanced stages, tumor-associated macrophages (TAMs), myeloid-derived suppressor cells, and regulatory T cells exert immunosuppressive capabilities, inducing angiogenesis and even metastasis^12^. Chronic alcohol consumption influences immune cells in the liver, such as Kupffer cell activation^13^, iNKT cell accumulation^14, 15^, neutrophil infiltration^15, 16^, inhibition of NK cell activity^17^, and dendritic cell (DC) function^18^. Moreover, chronic alcohol consumption can also induce altered expression of hepatic cytokines, including IL-1β, IL-6, IL-10, TNF-α, and IL-22^14, 19, 20^. HCC is a typical inflammation-induced cancer. Clinical studies have reported higher expression of IL-1β, IL-6, IL-10, and IL-22 in HCC patients, thus promoting HCC^21–23^. Given that immune cells play key roles in HCC development, the effect of chronic alcohol consumption on hepatic immune status during HCC has not been well described. In addition, the immune mechanisms by which alcohol consumption modulates HCC progression remain unclear.

In our mouse alcohol-DEN-HCC model, chronic alcohol consumption exacerbated inflammation and fibrosis and promoted EMT and HCC. Moreover, Chronic alcohol consumption decreased levels of antitumor CD8^+^ T cells but increased TAMs. Our data show that ethanol consumption alters intrahepatic immune microenvironment and pathological management, thereby affecting HCC development.

## Results

### Chronic alcohol consumption promotes DEN-initiated tumorigenesis

**Figure 1:**
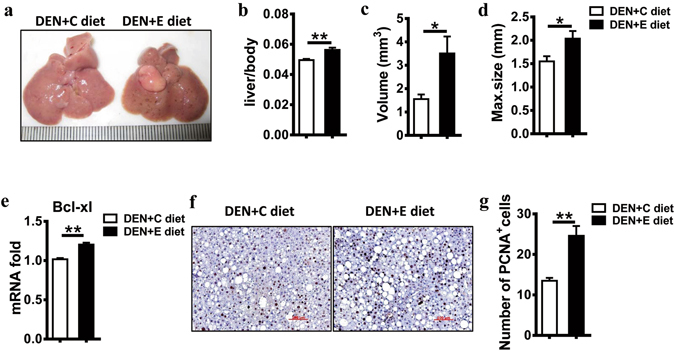
Alcohol consumption promotes DEN-initiated hepatocarcinogenesis in male mice. (**a**) Representative images showing HCC development in male mice given DEN with the control diet (DEN + C diet) or DEN with ethanol feeding (DEN + E diet). (**b**) Liver/body weight ratio was compared with DEN + C diet and DEN + E diet mice. Data are shown as the means ± SEM (n = 8 for DEN + C diet, n = 10 for DEN + E diet). **P < 0.01. (**c**) Tumor volume was compared with DEN + C diet and DEN + E diet mice. Data are shown as the means ± SEM (n = 10 for DEN + C diet, n = 8 for DEN + E diet). *P < 0.05. (**d**) Greatest tumor diameter was compared with DEN + C diet and DEN + E diet mice. Data are shown as the means ± SEM (n = 10 for DEN + C diet, n = 8 for DEN + E diet). *P < 0.05. (**e**) Bcl-xl mRNA expression in livers were analyzed by Q-PCR in DEN + C diet and DEN + E diet mice. Gene expression level was normalized to housekeeping control β-actin. Data are shown as the means ± SEM (n = 3 for DEN + C diet, n = 6 for DEN + E diet). **P < 0.01. (**f**) Cell proliferation levels assessed through PCNA staining (original magnification, 200×. Scale bar, 100 μm). (**g**) The number of PCNA-positive cells per field were compared with DEN + C diet and DEN + E diet mice. Data are shown as the means ± SEM (n = 4 for DEN + C diet, n = 5 for DEN + E diet). **P < 0.01.

To study the effect of chronic alcohol consumption on tumor progression, the procedure was performed as described (Supplemental Fig. S1). The mice fed alcohol for 2.5 months exhibited more HCCs per liver than did the pair-fed mice (Fig. 1a). Moreover, alcohol consumption significantly increased the liver/body weight ratios (Fig. 1b), tumor volume (Fig. 1c), and tumor maximum diameter (Fig. 1d). Alcohol consumption markedly promoted the expression of Bcl-xl (Fig. 1e) and PCNA (Fig. 1f,g) in the DEN-treated livers. These data suggest that chronic alcohol consumption promotes DEN-initiated tumorigenesis.

### Chronic alcohol consumption increases tumor-associated macrophage infiltration

**Figure 2:**
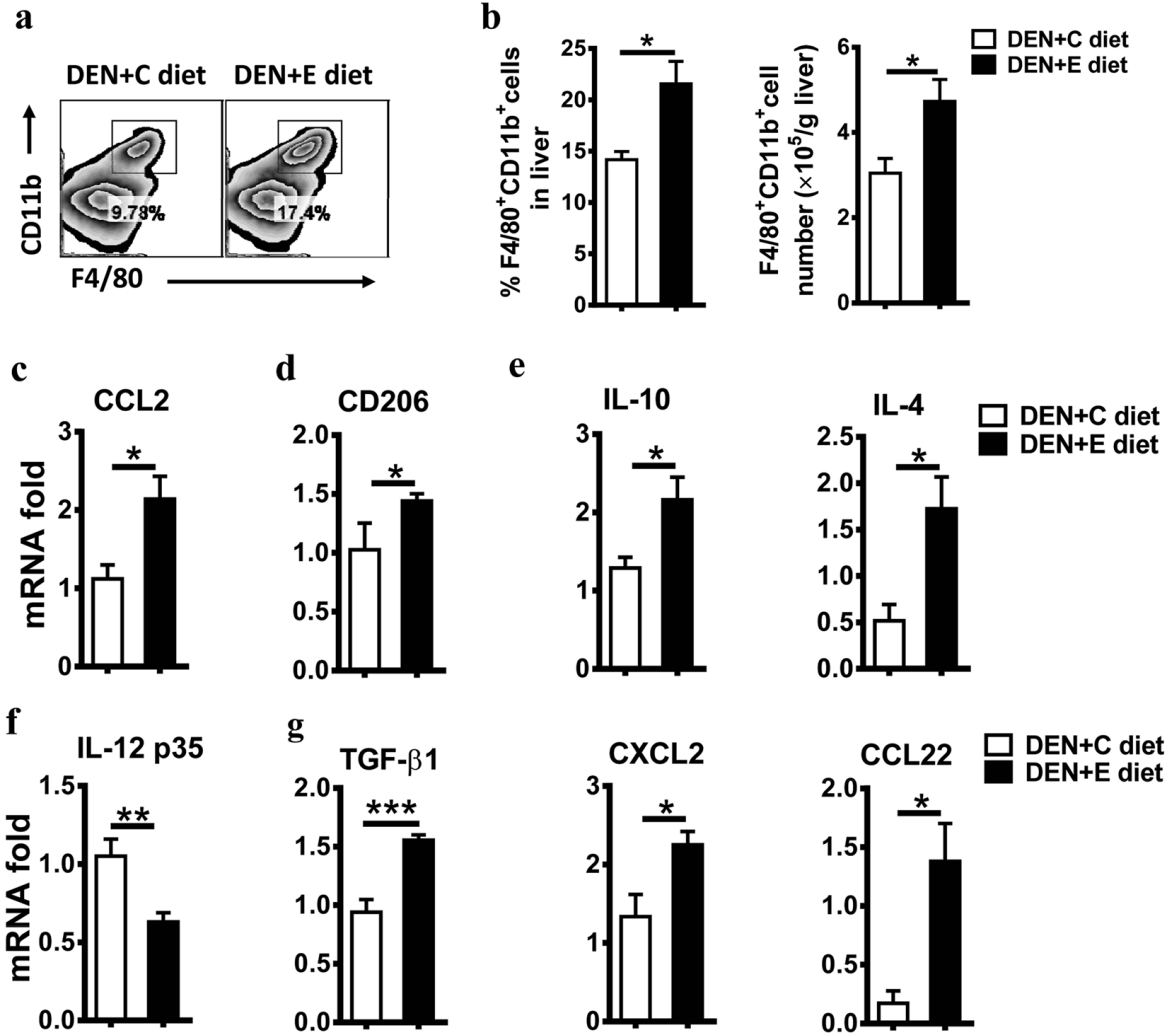
Alcohol consumption increases tumor-associated macrophage infiltration and favors M2 macrophage formation in DEN-initiated tumorigenesis. (**a**) The frequency of CD11b^+^F4/80^+^ cells in livers was evaluated via flow cytometry (FACS) in DEN + C diet and DEN + E diet mice. (**b**) Statistical analysis of the percentage and absolute number of CD11b^+^F4/80^+^ cells in livers were performed in DEN + C diet and DEN + E diet mice. Data are shown as the means ± SEM (n = 4 for DEN + C diet, n = 5 for DEN + E diet). *P < 0.05. (**c**) Analysis of hepatic monocytes recruitment associated gene mRNA levels in DEN + C diet and DEN + E diet mice, as determined by Q-PCR. All gene expression levels were normalized to housekeeping control β-actin. Data are shown as the means ± SEM (n = 4 for DEN + C diet, n = 6 for DEN + E diet). *P < 0.05. (**d**–**g**) mRNA levels of CD206, IL-10, IL-4, IL-12 p35, TGF-β, CXCL2, CCL22 in DEN + C diet and DEN + E diet mice were determined by Q-PCR. Data are shown as the means ± SEM (n = 3–4 for DEN + C diet, n = 4–6 for DEN + E diet). *P < 0.05. **P < 0.01. ***P < 0.001.

Hepatocarcinogenesis is a multifactorial and dynamic event that involves inflammation, fibrosis, and epithelial-mesenchymal transition (EMT), in which immune cells and immune factors are known to play important roles. Therefore, we examined the changes of immune status induced by chronic alcohol consumption in the DEN-treated liver. Firstly, the frequency and number of macrophages in the liver were higher in the DEN + E diet group than those in the DEN + C diet group (Fig. 2a,b). In accordance, higher mRNA expression of CCL2 were found in the DEN + E diet group (Fig. 2c), which could recruit monocytes into the liver. Moreover, Hepatic CD206 expression was elevated in the DEN + E group (Fig. 2d). This indicated that macrophages after chronic alcohol consumption were prone to be alternatively activated M2 macrophages. As expected, the expression of M2-polarization factors IL-4 and IL-10 dramatically increased in the DEN + E group (Fig. 2e), whereas M1-polarization factor IL-12 expression was lower in the DEN + E group (Fig. 2f). Besides, M2-associated protumor genes in the liver were elevated after chronic alcohol consumption, including TGF-beta, CXCL2 and CCL22 (Fig. 2g). These data indicate that chronic alcohol consumption promotes the accumulation of macrophages into the liver and M2 polarization.

### Chronic alcohol consumption reduces antitumor CD8^+^ T cells

**Figure 3:**
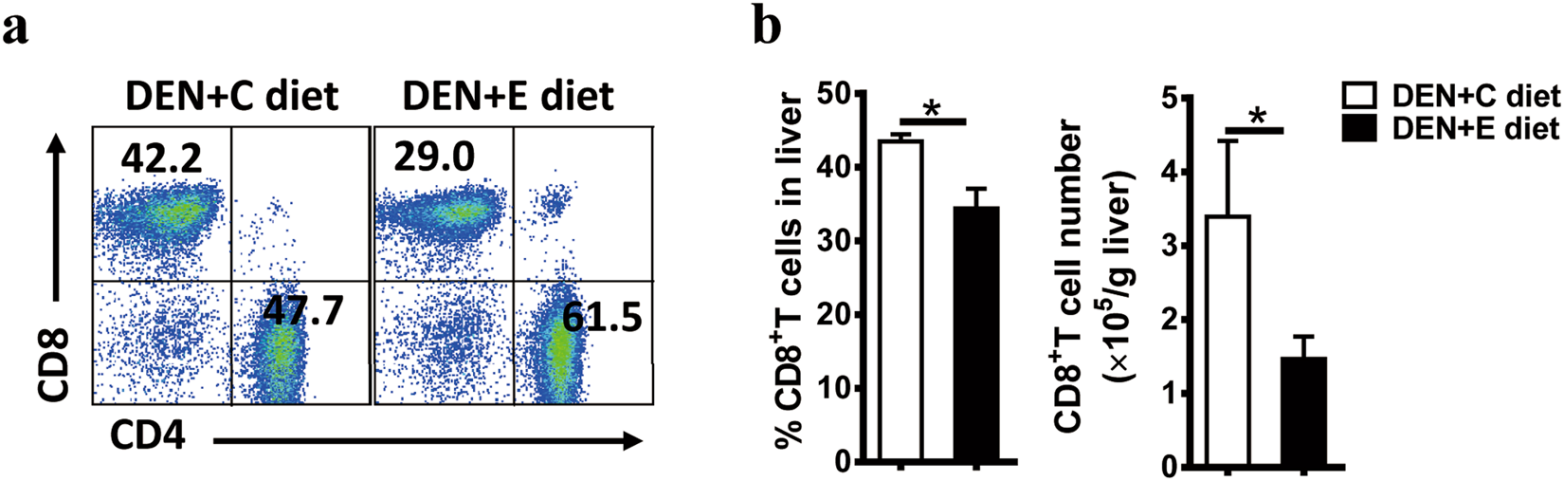
Alcohol consumption reduces CD8^+^ T cells in the liver in DEN-initiated tumorigenesis. (**a**) CD3^+^CD4^+^ or CD3^+^CD8^+^ T cell frequency in livers were evaluated by using FACS. (**b**) Statistical analysis of the percentage and absolute number of CD3^+^CD8^+^ T cells in livers were performed in DEN + C diet and DEN + E diet mice. Data are shown as the means ± SEM (n = 4 for DEN + C diet, n = 6 for DEN + E diet). *P < 0.05.

The roles of chronic alcohol consumption in antitumor immunity were also explored after accessing the protumor immunity. CD8^+^ T cells and NK cells are essential antitumor immune cells. As shown in Fig. 3, chronic alcohol consumption decreased the frequency and number of CD8^+^ T cells in the DEN-treated liver (Fig. 3a–c). However, chronic alcohol consumption had no effect on the frequency and number of NK cells in the liver (data not shown). These data suggest that chronic alcohol consumption weakens antitumor immunity in DEN-initiated tumorigenesis.

### Chronic alcohol consumption aggravates inflammation

**Figure 4:**
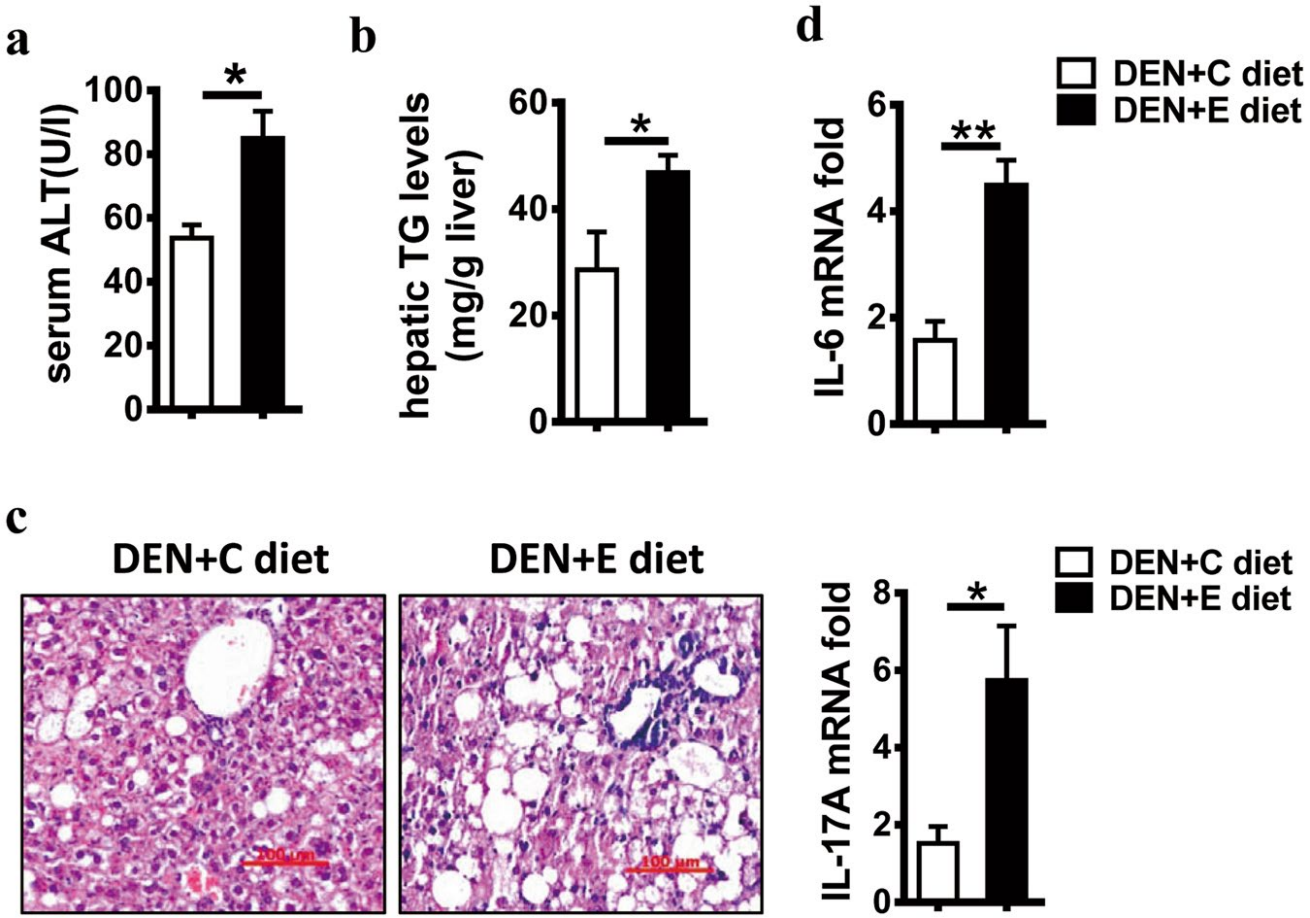
Alcohol consumption enhances the level of inflammation in DEN-initiated hepatocarcinogenesis. Liver injury and steatosis in DEN + C diet and DEN + E diet mice were evaluated by (**a**) serum alanine aminotransferase (ALT) levels and (**b**) hepatic triglyceride (TG) levels as well as (**c**) hematoxylin and eosin staining (H&E) (original magnification, 200 ×. Scale bar, 100 μm) and (**d**) relative mRNA expression of IL-6, IL-17A in livers, as determined by Q-PCR. Data are shown as the means ± SEM (n = 3–4 for DEN + C diet, n = 4–6 for DEN + E diet). *P < 0.05. **P < 0.01.

Given that HCC is a typical inflammation-related tumor, we wondered whether chronic alcohol consumption would exacerbate hepatic inflammation. Compared with the pair-fed mice in the DEN + C group, the mice in the DEN + E group exhibited significantly higher ALT (Fig. 4a) and TG levels (Fig. 4b) and displayed more fat vacuoles in the liver (Fig. 4c), indicating more severe liver damage and pan-lobular steatosis in the DEN + E group. Besides, as shown in Fig. 4d, the hepatic mRNA levels of HCC-promoting inflammatory factors, including IL-6 and IL-17A were markedly increased in the DEN + E group compared with the pair-fed group. These data suggest that chronic alcohol consumption aggravated inflammation level in the process of DEN-induced hepatocarcinogenesis.

### Chronic alcohol consumption exacerbates fibrosis and promotes EMT

**Figure 5:**
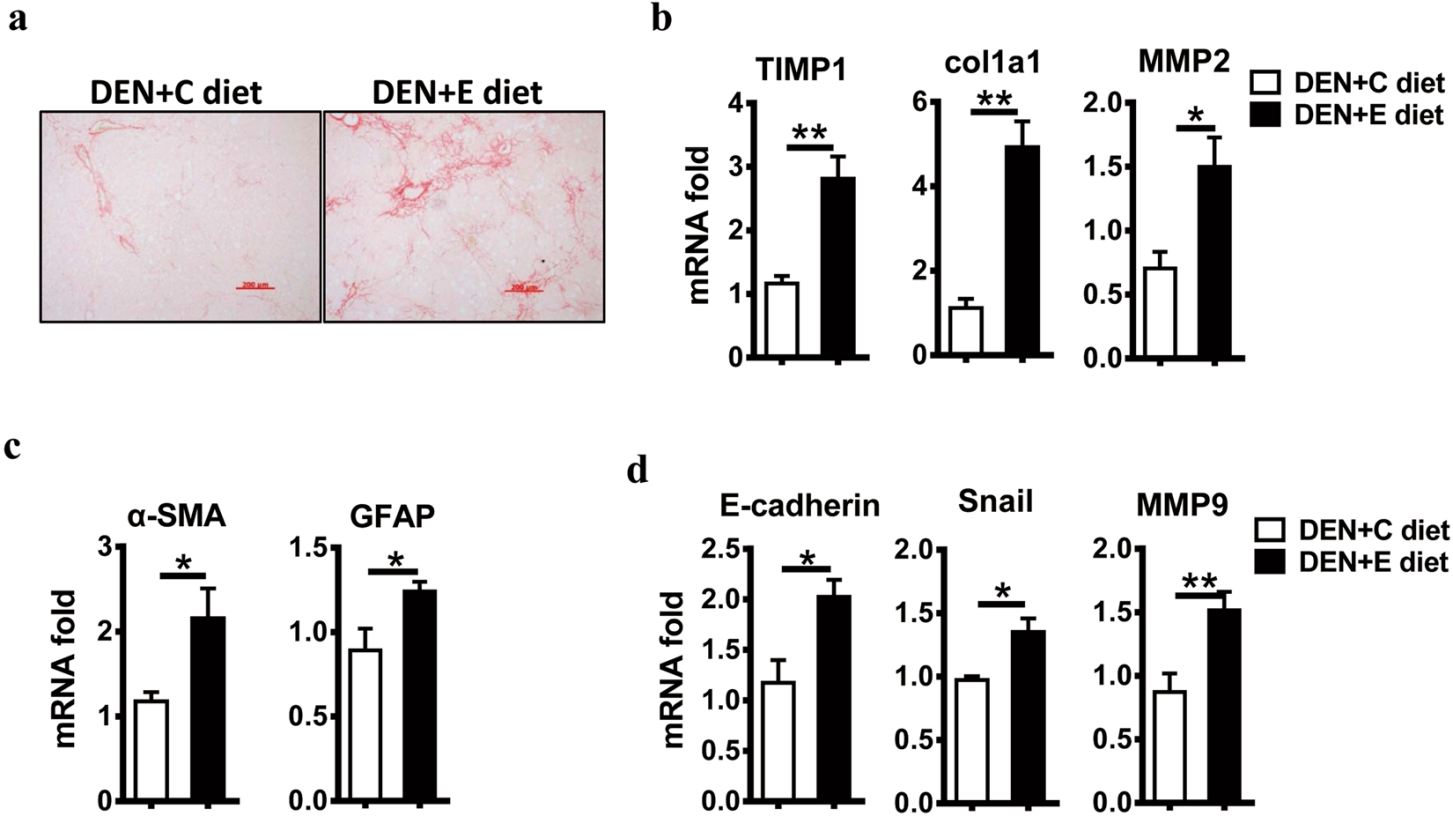
Alcohol consumption promotes liver fibrosis and epithelial-mesenchymal transition in DEN-initiated tumorigenesis. (**a**) Sirius red staining of sections (original magnification, 200 ×. Scale bar, 200 μm) were performed in DEN + C diet and DEN + E diet treated-livers. (**b**) Fibrosis marker gene expression levels in DEN + C diet and DEN + E diet were analyzed by Q-PCR. Data are shown as the means ± SEM (n = 4 for DEN + C diet, n = 6 for DEN + E diet). *P < 0.05. **P < 0.01. (**c**) HSC activation marker gene expression levels in DEN + C diet and DEN + E diet were determined by Q-PCR. Data are shown as the means ± SEM (n = 4 for DEN + C diet, n = 4–5 for DEN + E diet). *P < 0.05. (**d**) Hepatic mRNA levels of E-cadherin, Snail, MMP9 in DEN + C diet and DEN + E diet mice were analyzed via Q-PCR. Data are shown as the means ± SEM (n = 4 for DEN + C diet, n = 6 for DEN + E diet). *P < 0.05. **P < 0.01.

Human HCCs occur primarily in the setting of clinical fibrosis or cirrhosis. Considering the worse steatohepatitis in the DEN + E group, we wondered whether chronic alcohol consumption played a role in hepatic fibrosis. Notably, compared with pair-fed mice in the DEN + C group, mice in the DEN + E group showed more fibrous septa in liver sections, as visualized by Sirius red staining (Fig. 5a). Moreover, the mRNA expression of the fibrosis-related genes Col1A1, MMP2, and TIMP1 in the liver were higher in the DEN + E group than in the DEN + C group (Fig. 5b). Activated hepatic stellate cells (HSCs) are the main collagen-producing cells in liver fibrosis^24^. α-SMA and GFAP are markers of HSC activation^24, 25^. The levels of α-SMA and GFAP were markedly greater in the DEN + E group compared to the DEN + C group (Fig. 5c). In addition, EMT is increasingly recognized as a crucial driver of cell plasticity and contributes to therapeutic resistance, tumor recurrence, and metastatic progression^26^. Chronic alcohol consumption significantly increased the expression of the EMT-related genes snail, E-cadherin, and MMP9 in the livers of the DEN + E group (Fig. 5d). These data suggest that chronic alcohol consumption promotes liver fibrosis and EMT in the process of hepatocarcinogenesis.

## Discussion

**Figure 6:**
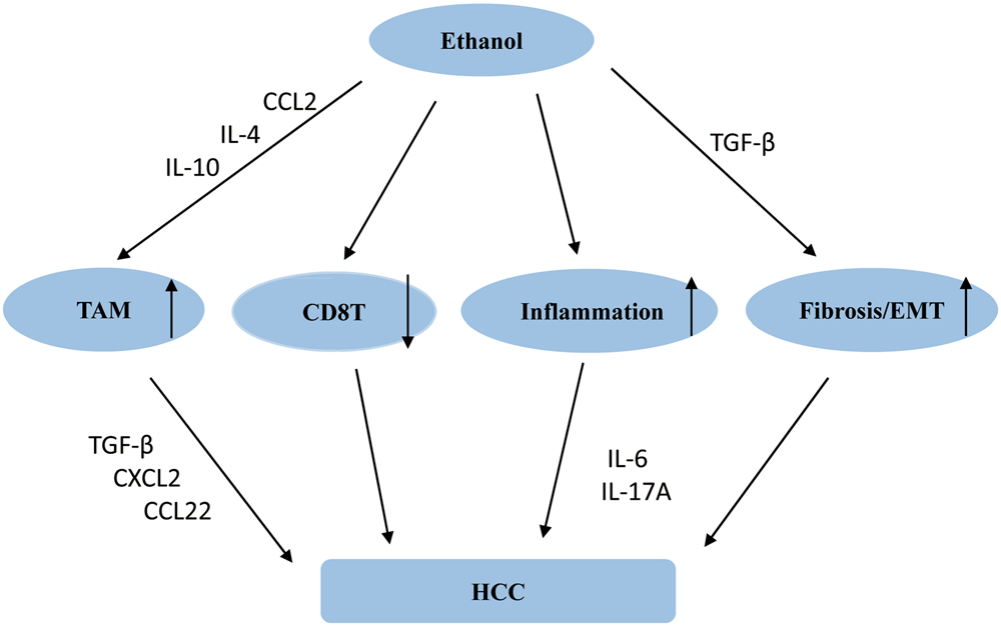
Multifactorial effects of ethanol promotion on DEN-initiated hepatocellular carcinoma. Alcohol consumption accelerated DEN-initiated HCC via many aspects. Firstly, alcohol consumption increased hepatic IL-4, IL-10, CCL2 expression, which in turn recruited monocytes into liver and polarization into M2-phenotype. Increased hepatic TGF-β, CXCL2, CXCL22 expression promoted HCC progression. Secondly, alcohol consumption reduced CD8^+^ T cells in the liver, which exhibited antitumor activity in existing study. Besides, alcohol consumption leaded a higher inflammation response in the liver, including increasing IL-6, IL-17A, which displayed a promotion on HCC progression. Moreover, increased hepatic TGF-β expression after alcohol consumption facilitated fibrosis and EMT progress, which were associated with HCC occurrence.

Alcohol abuse is a significant risk factor for liver diseases, owing to changes in lifestyle, regardless of other factors. Epidemiological data have suggested that alcohol abuse contributes to HCC development^1, 2^. In the current study, we demonstrated that alcohol promotes HCC progress in mice via increasing tumor-associated macrophage infiltration, reducing antitumor CD8^+^ T cells, exacerbating fibrosis, and promoting EMT (Fig. 6).

The effects of chronic alcohol consumption on HCC progression vary in different animal models. In accordance with Elizabeth Brandon-Warner and Aditya Ambade’s studies^8, 9^, chronic alcohol consumption promoted DEN-induced HCC progression in our model. However, Maotai liquor ameliorates DEN-induced hepatocarcinogenesis^7^. The factors contributing to these contradictory outcomes are likely to be complex and to include factors such as the carcinogen class (chemical or biological), time and dose of DEN injection, ethanol category, ethanol feeding concentration, and duration.

The tumor milieu contains many macrophages, which are extremely heterogeneous and are divided systematically into two main classes resembling the Th1/Th2 dichotomy^27^. Chemokines are crucial factors for monocytes migration into local tissue. Chronic alcohol consumption increased hepatic CCL2 expression, which promote macrophages accumulation into the liver^28^. TAMs tend to have a protumor M2-like phenotype. Alcohol abuse increases IL-4 and IL-10 levels as well as TGF-beta expression in the liver, thus inducing alternatively activated M2 macrophages. Moreover, CD206, a marker for M2 macrophages, also showed a higher expression level in the DEN + E group, consistent with findings from a recent report^8^. This information indicated that chronic alcohol abuse promotes a shift to M2 macrophages.

CD8^+^ T cells are primary antitumor immune cells. Data have confirmed that the numbers and activity of CD8^+^ T cells decrease in the advanced tumor microenvironment^29^. The numbers of CD8^+^ T cells in the liver after chronic alcohol consumption were significantly reduced in DEN-initiated hepatocarcinogenesis. The study from the comparison between patients with alcoholic cirrhosis and a healthy control identifies that ethanol could induce the apoptosis of CD8^+^ T cells^30^. Ethanol-induced apoptosis and higher TGF-β levels may count for CD8^+^ T cells decrease after chronic alcohol consumption in DEN-initiated hepatocarcinogenesis.

HCC is a typical inflammation-associated cancer. Chronic alcohol consumption leads to higher inflammation level in DEN-treated livers. IL-6 and IL-17A were up-regulated in DEN + E group, which are proved to promote HCC progression^31, 32^. Liver fibrosis is a consequence of the wound-healing response to virtually all forms of chronic liver injury. EMT involves modifying the adhesion molecules expressed by the cell, thus causing cells to obtain migratory and invasive abilities, and it is a significant driver during tumor progression^33^. Herein, we found that the markers of fibrosis and EMT in the DEN + E group showed higher expression than did the DEN + C group. TGF-β signaling has been shown to play an important role in fibrosis and EMT^34, 35^. As shown in Fig. 2. G, hepatic TGF-β expression was observably up-regulated after chronic alcohol consumption. These data suggest that chronic alcohol consumption activates fibrosis and EMT signaling via TGF-β in the liver, thereby contributing to HCC progression.

In summary, alcohol consumption promotes the development of HCC. Our data suggest that immune elements, including CD8^+^ T cells, macrophages as well as chemokines and cytokines, are changed by alcohol and are involved in the pathology of chronic alcohol consumption, promoting HCC in a DEN-initiated HCC mouse model. Future studies and clinical research should focus on immune cells, cytokines, and chemokines in hepatocarcinogenesis in the context of chronic alcohol consumption.

## Methods

### Materials

Diethylnitrosamine (DEN) was purchased from Sigma-Aldrich (St. Louis, MO). Ethanol was purchased from Sinopharm (Shanghai, China). An assay kit to measure alanine aminotransferase (ALT) was purchased from Rong Sheng (Shanghai, China). An assay kit to test triglyceride (TG) levels was purchased from Changchun Huili (Jilin, China). TRIzol reagent was purchased from Invitrogen (Carlsbad, CA). Antibodies against mouse proliferating cell nuclear antigen (PCNA) (ZSGB-BIO, China) were purchased for immunohistochemistry analysis.

### Animal Assurances

Male C57BL/6 mice (6–8 weeks old) were purchased from the Shanghai Laboratory Animal Center at the Chinese Academy of Sciences (Shanghai, China). All mice were maintained in specific-pathogen-free and temperature-controlled microisolator cages with a 12-h light/dark cycle. All the procedures regarding animal handling were performed in accordance with the animal care regulations of the University of Science and Technology of China. All the animal experiment protocols were approved by the Local Ethics Committee for Animal Care and Use at University of Science and Technology of China.

### Animal Experiments

Mice (approximately 2 months old) were weighed and injected with DEN (40 mg/kg body weight [BW] intraperitoneally [i.p.]) dissolved in saline every 4 days for a total of 10 times. Then, mice (approximately 6.5 months old) were randomly divided into two groups (Supporting Information Fig. 1A): ethanol groups were fed a liquid diet containing 5% ethanol (TROPHIC, Nantong, China) for 2.5 months, whereas control groups were pair-fed a control diet (TROPHIC, Nantong, China) for 2.5 months.

Mice were weighed and sacrificed after 7 months of DEN treatment. Livers were excised, weighed, and examined for visible lesions, and representative liver tissues (5 to 7 mm) were taken from the same lobes. Tissue samples were either snap frozen in liquid nitrogen and stored prior to analysis or fixed in 12% neutral and acidic buffered formalin (at least 36 hours) prior to histological processing. The remaining liver tissue samples were used for isolating liver mononuclear cells (MNCs).

### Alanine Liver Transaminase Activity and Triglyceride Content Analysis

Serum alanine aminotransferase (ALT) was detected using an automated Chemray 240 clinical analyzer (Rayto, Shenzhen, China). Total hepatic lipid extraction was performed according to the operation manual. The TG content was tested using a commercial diagnostic kit (Changchun Huili, Jilin, China).

### Hematoxylin & Eosin (H&E) and Picrosirius Red Staining

For histological analysis, liver tissues were fixed in 12% acidic buffered formalin, and paraffin-embedded liver samples were sliced into 6-µm-thick sections, deparaffinized, dehydrated, and stained with H&E. For fibrosis analysis, liver tissues were fixed in 12% neutral buffered formalin and embedded in paraffin. Tissues were affixed to slides, deparaffinized, and stained with Sirius red (0.1%). All the slices described above were examined under light microscopy.

### Immunohistochemical Staining

Liver tissues were fixed in 12% neutral buffered formalin to observe liver slides. For PCNA staining, liver slides were stained with a 1:100 dilution of an anti-PCNA (ZSGB-BIO, Beijing, China) primary antibody overnight, and this was followed by a polymer HRP detection system (ZSGB-BIO, Beijing, China). Brown stains were developed using a 3,3-diaminobenzidine kit (Vector Laboratories, Burlingame, CA).

### RNA Isolation and Quantitative RT-PCR (Q-PCR)

Total RNA was extracted from liver tissue using TRIzol reagent (Invitrogen, Carlsbad, CA, USA) according to the manufacturer’s instructions. cDNA was synthesized from random hexamers and reverse-transcribed using commercial reverse-transcription kits (Invitrogen). Gene expression levels were measured with Q-PCR using the commercially available SYBR Premix ExTaq (TaKaRa Biotechnology, Dalian, China) in a Corbett Rotor-Gene 3000 real-time PCR system (Corbett Research) according to the manufacturer’s instructions. Data were analyzed using the △△Ct method as previously described^36^. Fold changes in gene expression were calculated relative to the housekeeping gene beta-actin. The primer sequences are provided in Table S1.

### Liver Mononuclear Cell Isolation

Liver MNCs were isolated as previously described. Briefly, livers were passed through a 200-gauge stainless steel mesh. Cells were resuspended in 42% Percoll (Gibco BRL) and then centrifuged at 1260 g for 30 min at room temperature. The pellets were resuspended with red blood cell lysis buffer and washed with PBS. MNCs were used for cellularity and flow cytometric (FCM) analyses.

### Flow Cytometry and Antibody Staining

The fluorescence-labeled monoclonal antibodies were as follows: FITC-anti-CD3, PerCp-Cy5.5-anti-CD8, APC-Cy7-anti-CD11b, PerCp-Cy5.5-anti-F4/80, APC-anti-CD4, and PE-Cy7-anti-CD45 were purchased from Biolegend (San Diego, CA, USA); FITC-anti-CD11b was from BD Biosciences (San Jose, CA, USA); and PerCp-Cy5.5-anti-F4/80 was from eBioscience (San Diego, CA, USA). MNCs were stained with the indicated antibodies as previously described^37^. Data were collected on a FACSCalibur flow cytometer (BD Biosciences) and analyzed using FlowJo 7.6.1 (Tree Star, Ashland, OR).

### Statistical Analysis

All data were analyzed using Student’s t test and are expressed as the means ± standard error of the mean (SEM). P < 0.05 was considered statistically significant. Calculations were made by using GraphPad Prism version 6.0 (GraphPad Software, Inc., San Diego, CA).

## Electronic supplementary material

Supplement SR-Yan GX-alcohol-HCC
