# Supplementary material for: Chronic Alcohol Consumption Promotes Diethylnitrosamine-Induced Hepatocarcinogenesis via Immune Disturbances
Source: Sci Rep. 2017 May 31;7:2567. doi: 10.1038/s41598-017-02887-7 (PMC5451469; doi:10.1038/s41598-017-02887-7)
Supplement: Supplementary file 1 — Supplement SR-Yan GX-alcohol-HCC [file 41598_2017_2887_MOESM1_ESM.pdf]

Chronic Alcohol Consumption Promotes Diethylnitrosamine-Induced Hepatocarcinogenesis via Immune Disturbances

Guoxiu Yan, Xuefu Wang, Cheng Sun, Xiaodong Zheng, Haiming Wei, Zhigang Tian, and Rui Sun

Inventory of Supplementary Information

Supplementary Figure 1. Protocol used for studying the promotion of DEN-induced HCC by ethanol.

Supplementary Table 1. Primer sequences for genes.

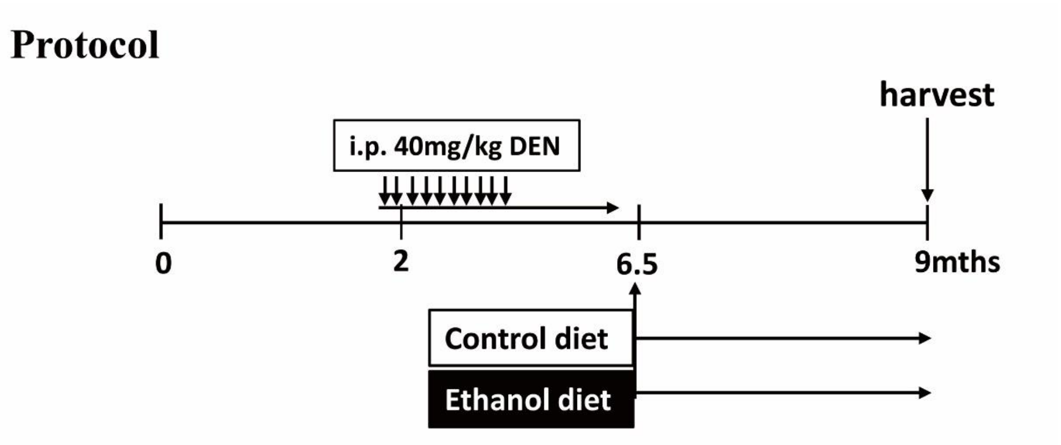

Supplementary Figure 1. Protocol used for studying the promotion of DEN-induced HCC by ethanol. Male mice (approximately 2 months old) were weighed and injected with DEN (40 mg/kg body weight [BW] intraperitoneally [i.p.]) dissolved in saline every 4 days for a total of 10 times. Then, mice (approximately 6.5 months old) were randomly divided into two groups: ethanol groups were fed a liquid diet containing 5% (V/V) ethanol for 2.5 months, whereas control groups were pair-fed a control diet for 2.5 months. Mice were weighed and sacrificed after 7 months of DEN treatment. The description in detail of experiment was seen in material and methods.

RT-PCR set	Forward primer	Reverse primer
Bcl-xl	TGAGCAGGTAGTGAATGAAC	TAGGTGGTCATTCAGATAGG
DGAT	TGGTGTGTGGTGATGCTGATC	GCCAGGCGCTTCTCAA
TIMP1	CTTGGTTCCCTGGCGTACTC	ACCTGATCCGTCCACAAACAG
col1a1	GAAACCCGAGGTATGCTTGA	GACCAGGAGGACCAGGAAGT
MMP2	TTTGCTCGGGCCTTAAAAGTAT	CCATCAAACGGGTATCCATCTC
α-SMA	CTGACAGAGGCACCACTGAA	GAAGGAATAGCCACGCTCAG
GFAP	AGAAAACCGCATCACCATTC	TCACATCACCACGTCCTTGT
E-cadherin	ACTGTGAAGGGACGGTCAAC	GGAGCAGCAGGATCAGAATC
snail	CCACTGCAACCGTGCTTTT	TCTTCACATCCGAGTGGGTTT
MMP9	ACTCACACGACATCTTCCAG	AGAAGGAGCCCTAGTTCAAG
CD206	CAGGTGTGGGCTCAGGTAGT	TGTGGTGAGCTGAAAGGTGA
IL-4	AGATGGATGTGCCAAACGTCCTCA	AATATGCGAAGCACCTTGGAAGCC
IL-10	GGTTGCCAAGCCTTATCGGA	ACCTGCTCCACTGCCTTGCT
TGF-beta 1	AAGTTGGCATGGTAGCCCTT	GCCCTGGATACCAACTATTGC
IL-12 p35	AAGACATCACACGGGACCAAA	CAGGCAACTCTCGTTCTTGTGTA
IL-6	GATGCTACCAAACTGGATATAATC	GGTCCTTAGCCACTCCTTCTGTG
IL-17A	GCTCCAGAAGGCCCTCAGA	AGCTTTCCCTCCGCATTGA
CCL2	GTTGGCTCAGCCAGATGCA	AGCCTACTCATTGGGATCATCTTG
CCL22	ACTTCAGACCTCCGATGCA	TGTAGTCCTGGCAGCAGATACT
CXCL2	CTCTCAAGGGCGGTCAAAAAGTT	TCAGACAGCGAGGCACATCAGGTA

Supplementary Table 1. Primer sequences for genes.
Detailed primer information for the mouse genes analyzed by Q-PCR.
